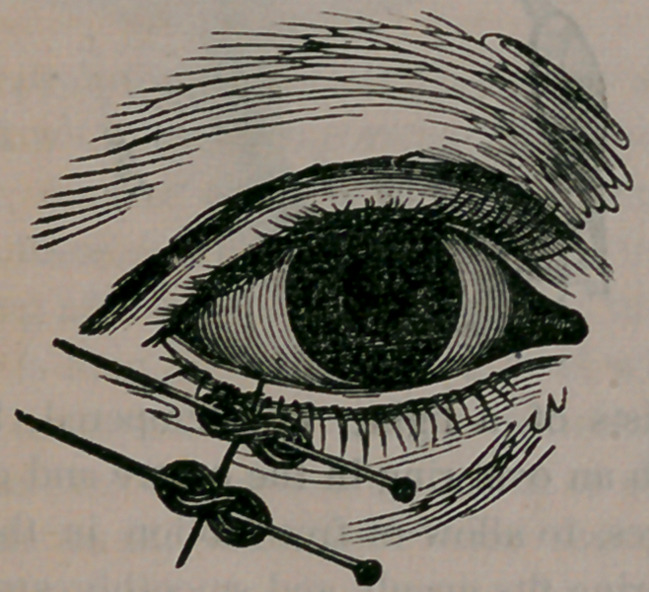# Ectropium

**Published:** 1875-04

**Authors:** 


### ECTROPIUM.

This is an affection of the eyelids, characterised by an eversion or exposure of the lining membrane of the lids.

It exists in various degrees; sometimes only a small portion of the *conjunctiva* or lining membrane is exposed, while at others, the entire lid is everted, causing a painful and unsightly appearance of the parts, the eyes appearing entirely surrounded by a red, inflamed membrane, from which the tears are continually dripping upon the cheek, while the eyes appear somewhat congested and swimming in tears. This exposure of the delicate *conjunctiva*, soon produces a chronic inflammation of that membrane, granular lids, enlargement, hardening and ulceration of the parts; and, from the increased exposure of the eye to the atmosphere, even inflammation and consequent dimness of the cornea ensues.

We often see *ectropium* in aged people, particularly if they have been subjects of inflammation of the conjunctiva. Children, too, who have had a purulent inflammation of the eyes, with consequent swelling of the lids, often have eversion of the lids after the primary disease has subsided. Burns, wounds or ulcers upon or near the lids, when healed are apt to contract the skin upon the outside, and so evert the lids. We have seen it produced in children from the healing of a protracted *eczema*, or skin disease.

Aside from the unsightly appearance of the eyes when affected with this disease, the affection demands our attention; for if it be not remedied, it will eventually ruin the eye.

We give here an illustration of ectropium of the lower lid, showing the eversion of the lid and consequent exposure of the delicate conjunctiva and eye to the irritating influences of the atmosphere. The lines marked upon the lid, show the direction the surgeon's knife takes in excising a portion of the lid, of proper size and shape to cause, when the edges of the wound are united, a restoration of the parts to their natural position, as is illustrated in the cut which follows:

This is only *one* of the plans adopted by the surgeon in remedying difficulties of this nature. When the *ectropium* is produced by scars from scalds, wounds or burns, pieces of integument or skin is often transplanted in order to give the contracted lid its proper position. But from whatever cause such a condition of affairs may arise, the sufferer can rely upon prompt relief through the skillful surgeon's hand.

## Figures and Tables

**Figure f1:**
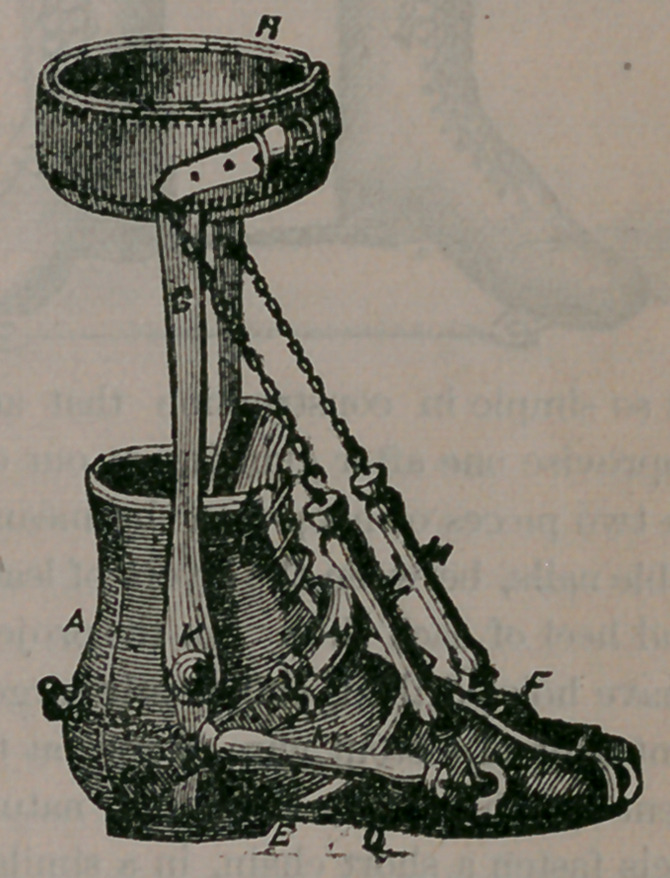


**Figure f2:**